# Pitfalls associated with the therapeutic reference pricing practice of asthma medication

**DOI:** 10.1186/1471-2466-12-35

**Published:** 2012-07-20

**Authors:** Zoltan Kalo, Zsolt Abonyi-Toth, Zoltan Bartfai, Zoltan Voko

**Affiliations:** 1 Department of Health Policy and Health Economics, Institute of Economics, Faculty of Social Sciences, Eötvös Loránd University, Budapest, Hungary; 2 Syreon Research Institute, Budapest, Hungary; 3 RXPress Ltd, Budapest, Hungary; 4 Elisabeth Teaching Hospital Sopron, Department of Pulmonology, Sopron, Hungary; 5 Health Economics Research Centre, Institute of Economics, Faculty of Social Sciences, Eötvös Loránd University, Pázmány Péter sétány 1/a, Budapest, H-1117, Hungary

## Abstract

**Background:**

Therapeutic reference pricing (TRP) based on the WHO daily defined dose (DDD) is a method frequently employed for the cost-containment of pharmaceuticals. Our objective was to compare average drug use in the real world with DDD and to evaluate whether TRP based on DDD could result in cost savings on maintenance medication and the total direct health expenditures for asthma patients treated with Symbicort Turbuhaler (SYT) and Seretide Diskus (SED) in Hungary.

**Methods:**

Real-world data were derived from the Hungarian National Health Insurance Fund database. Average doses and costs were compared between the high-dose and medium-dose SYT and SED groups. Multiple linear regressions were employed to adjust the data for differences in the gender and age distribution of patients.

**Results:**

27,779 patients with asthma were included in the analysis. Average drug use was lower than DDD in all groups, 1.38-1.95 inhalations in both SED groups, 1.28-1.97 and 1.74-2.49 inhalations in the medium and high-dose SYT groups, respectively. Although the cost of SED based on the DDD would be much lower than the cost of SYT in the medium-dose groups, no difference was found in the actual cost of the maintenance therapy. No significant differences were found between the groups in terms of total medical costs.

**Conclusions:**

Cost-containment initiatives by payers may influence clinical decisions. TRP for inhalation asthma drugs raises special concern, because of differences in the therapeutic profile of pharmaceuticals and the lack of proven financial benefits after exclusion of the effect of generic price erosion. Our findings indicate that the presented TRP approach of asthma medications based on the daily therapeutic costs according to the WHO DDD does not result in reduced public healthcare spending in Hungary. Further analysis is required to show whether TRP generates additional expenditures by inducing switching costs and reducing patient compliance. Potential confounding factors may limit the generalisability of our conclusions.

## Background

The question of what proportion of total healthcare costs should be financed from public resources and what proportion should be spent on pharmaceuticals is under continuous debate in Hungary [1]. Before 2006, the proportion of the total drug cost financed from public resources was larger than the average for OECD countries. Furthermore, between 2000 and 2006, the increase in public pharmaceutical expenditures exceeded the annual rate of 17%. Therefore, the Hungarian National Health Insurance Fund (NHIF) applied a series of cost-containment measures to limit the growth rate of pharmaceutical spending [2,3].

One element of these measures was the extension of the therapeutic reference pricing (TRP) system, also known as the system of therapeutic reference classes. The method implies that the actual reimbursement for the drugs included in the same class (e.g. ATC class with four or five digits) is maximised in line with the average price of products, with the lowest daily therapeutic costs included in that class. The payer indirectly influences the drug price by defining the maximum reimbursement according to the preferred price, and manufacturers can reduce the price of non-referenced products to avoid high copayment. The group of referenced products with the lowest daily therapeutic costs should reach a proportion of 50% in the whole class, measured by the number of days of treatment (DOT).

Several technical objections may be raised against the implementation of the TRP system [4]. First, when including drugs that contain different active ingredients in the same reference class, decision-makers usually fail to take into account significant differences in the indications of the drugs, despite their similar mechanisms of action. Second, even if the indications of drugs in a TRP group are the same, there may still be some differences in their efficacy, tolerability and adverse event profiles in the entire patient population or in a subgroup of patients. Because different substances in the same class can be metabolised in different ways, they may show different drug interaction profiles. In such cases, the optimal therapy for a certain group of patients may differ from the medication that is appropriate for most other patients. TRP for inhalation drugs raises special concern because, despite the identical route of administration, the different characteristics of the inhalation devices may lead to differences in efficacy (e.g. lung deposition). Finally, the establishment of therapeutically equivalent daily doses can be difficult in the case of TRP because comparisons can be made only according to the principles of evidence-based medicine based on direct comparative studies. Instead of this, the WHO DDD (average defined daily dose) is used as the basis for the equivalent dose by payers. Even the WHO, however, objects to the use of DDD in decision-making regarding efficacy, pricing and reimbursement [5].

Although concerns have been raised about the use of TRP from the perspective of drug innovation and industrial policy, these are usually offset by financial aspects because the widespread use of reference pricing can lead to massive savings in the pharmaceutical budget [6][7]. The impact of TRP is often mixed with savings from generic reference pricing (GRP), so it is difficult to determine how much of the saving from reference pricing is attributable to the increased utilisation of generics and generic price erosion. In some cases, when the impact of TRP is separated from GRP, TRP does not display clear savings in pharmaceutical expenditures [8].

The aim of our study was to evaluate the effects of TRP in the case of drugs used in the treatment of asthma. High-dose Seretide Diskus and Symbicort® Turbuhaler® with maximum reimbursement rates of 90% had already been reimbursed by the TRP system within the R03AK06 ATC group. During the period of the analysis no other original or generic products were included in this TRP group. The inclusion of the medium-dose products in this pricing system seems to be the next feasible step. In the high-dose class, TRP ensured an advantageous position for the Symbicort Turbuhaler on the basis of the WHO DDD because the price of a daily dose is 3.7% lower than the price of Seretide Diskus. TRP for a medium dose would make the Seretide Diskus the reference product because the price of a daily dose is 21.2% to 24.1% lower than the price of the two packaged forms of Symbicort.

There are several differences, however, between Seretide Diskus and Symbicort Turbuhaler regarding the indications for and the effects of the drugs. Symbicort Turbuhaler can also be used as maintenance and reliever therapy, unlike Seretide Diskus, which is appropriate only for maintenance therapy. This difference results from formoterol, the beta-agonist component of Symbicort. Unlike salmeterol, formoterol not only has a long-term effect but also a rapid bronchodilating effect [9]. Formoterol reaches its effect in three to four minutes, similarly to the widely-used reliever salbutamol [10].

Compared with salmeterol, formoterol has a possible advantage in its dose-dependent bronchoprotective effects on methacholine-induced bronchoconstriction [11]. The maximum daily dose of formoterol in the treatment of asthma is eight inhalations, or 36 mcg; however, for temporary regiments of a few days, 12 inhalations, or 54 mcg, are also allowed. The same cannot be said for salmeterol because its maximum daily dose is 100 mcg or 2x1 inhalation for all dosage forms of this product. This is because of the narrow therapeutic window, the systemic effects that appear above the maximal dose and the lack of further smooth muscle-relaxing effects above the maximal dose [12,13].

Taking into account the WHO DDD, it can be expected that the price of Symbicort Turbuhaler for patients on chronic therapy will be lower for the high-dose form and higher for the medium dose when compared with Seretide Diskus. Due to the rapid bronchodilating effects of Symbicort, the use of other relievers is expected to be lower in patients using Symbicort Turbuhaler than in those treated with Seretide Diskus.

Due to its wide therapeutic window and rapid reliever effects, formoterol allows for acute needs-based administration (see the SMART indication for Symbicort), which may reduce the number of exacerbations if the steroid component is administered at the appropriate time. Furthermore, the narrower therapeutic window of salmeterol could lead to a higher incidence of adverse events in patients exceeding the upper limit of the therapeutic window.

In our study we evaluated whether in the real world there was a difference in the costs of maintenance medication, attack-reliever therapies, treatment of exacerbations and total direct health expenditures between patients treated with Symbicort Turbuhaler and Seretide Diskus. We used real-world cost data from the NHIF for analysis.

## Methods

For the selection of patients, we used real-world outpatient care, inpatient care and drug utilisation databases (data on prescriptions filled in pharmacies) of the NHIF. IRB approval was not necessary for the retrospective analysis of aggregated patient records.

We included adult patients who were born before 1990, had filled a medium- or high-dose prescription of Symbicort (SYT) or Seretide Diskus (SED) in the first two months of 2008, and had filled a SYT or SED prescription at least twice in 2008 with 90% reimbursement of NHIF with an asthma ICD10 code (J45, J96). We excluded patients who filled prescriptions for both study drugs (i.e. those who were mixing SYT and SED or those who also used Seretide Evohaler) and those who filled different dosage forms in 2008 (Table 1).

**Table 1:** Classification of study drugs according to strength

**Name**	**Package form**	**Number of days of treatment**	**Strength**	**DTC**
Symbicort Forte Turbuhaler	1 × 60 doses	30	high dose	3.35
Seretide Diskus 50/500	1 × 60 doses	30	high dose	3.48
Symbicort Turbuhaler	1 × 60 doses	15	medium dose	3.57
Symbicort Turbuhaler	1 × 120 doses	30	medium dose	3.35
Seretide Diskus 50/250	1 × 60 doses	30	medium dose	2.64

DTC: daily therapeutic costs based on the WHO DDD (USD).

The National Health Insurance Fund provided us with data on the mean annual costs for 2008 for the following cost items by age group (18–30, 31–40, 41–50, 51–60, 61–70, >70 years), gender, maintenance drug type (SYT or SED) and dosage form (medium or high dose):

 1. units and public prices of maintenance antiasthmatic pharmaceutical therapies (SYT or SED),

 2. units and public prices of reliever medications (drugs containing salbutamol, terbutaline or fenoterole),

 3. units and public prices of medications used for exacerbation (containing ampicillin, amoxicillin, penicillin, tetracycline, trimetoprim and sulfamethoxazole, cefuroxime, cefamandol, cefaclor, cefprozil, cefotaxim, ceftazidim, ceftriaxon, cefixim, cefoperazone, ceftibuten, azithromycin, clarithromycin, roxithromycin, ofloxacin, ciprofloxacin, levofloxacin, moxifloxacin, or methylprednisolone).

 4. public prices of all filled prescription drugs,

 5. reimbursement for outpatient care required for asthma (the ICD code of the disease justifying the care being J45 or J96),

 6. reimbursement for all outpatient care,

 7. reimbursement for inpatient care required for asthma (the ICD code of the disease justifying the care being J45 or J96),

 8. reimbursement for all inpatient care.

We converted Hungarian forints into US dollars by employing the 2009 GDP PPP exchange rate published by the OECD (1 USD = 135 HUF).

We calculated the average daily inhalations of the maintenance drugs by dividing the total quantity of prescribed medications filled without the last prescription over the number of days between the first and last first prescriptions filled.

We did not receive data for the other comorbidities of the patients and assumed that there were no differences between the patient groups in this respect.

In the descriptive statistical analysis, we compared the inpatient costs, the outpatient costs, the costs of the maintenance medications, costs of the drugs used for exacerbation, costs of the reliever therapies and all other medication therapies, and the total costs in all four groups (high-dose SYT, medium-dose SYT, high-dose SED and medium-dose SED).

Next, we used multiple linear regressions to adjust the data for the differences in gender and age distribution of the patients receiving different maintenance treatments. Because only aggregate data were available, it was not possible to use linear regressions based on the traditional least-square method. Instead, we performed a regression analysis on the aggregated data using the metareg program of the STATA10 statistical program package [14]. This analysis can be considered as a way of standardising the mean differences in costs for both age and gender.

## Results

We analysed the data from a total of 27,799 asthma patients; 12,260 used Seretide Diskus, and 15,539 used Symbicort Turbuhaler. Table 2 shows the distribution of the study population according to age, gender and maintenance therapy. The proportion of the patients over 60 years of age was a few percentage points higher in all study groups among Seretide users than among Symbicort users.

**Table 2:** Distribution of the study population by age group, gender, maintenance therapy and strength of therapy

	**High dose therapy**				**Medium dose therapy**			
	**Male**		**Female**		**Male**		**Female**	
**Age group (year)**	**Seretide**	**Symbicort**	**Seretide**	**Symbicort**	**Seretide**	**Symbicort**	**Seretide**	**Symbicort**
18-30	93 (3.7%)	125 (7.6%)	100 (3.0%)	79 (3.5%)	364 (16.4%)	728 (16.5%)	321 (7.7%)	703 (9.7%)
31-40	176 (7.0%)	188 (11.4%)	195 (5.8%)	183 (8.2%)	295 (13.3%)	753 (17.1%)	355 (8.5%)	804 (11.1%)
41-50	249 (9.9%)	210 (12.8%)	424 (12.6%)	336 (15.0%)	305 (13.8%)	627 (14.2%)	556 (13.3%)	1 106 (15.3%)
51-60	654 (26.1%)	382 (23.2%)	1 008 (30.0%)	685 (30.6%)	451 (20.3%)	878 (19.9%)	1 100 (26.4%)	1 956 (27.0%)
61-70	679 (27.1%)	416 (25.3%)	842 (25.0%)	522 (23.3%)	423 (19.1%)	797 (18.1%)	954 (22.9%)	1 528 (21.1%)
71-X	654 (26.1%)	326 (19.8%)	795 (23.6%)	436 (19.5%)	380 (17.1%)	622 (14.1%)	887 (21.3%)	1 149 (15.9%)
Total	2 505 (100%)	1 647 (100%)	3 364 (100%)	2 241 (100%)	2 218 (100%)	4 405 (100%)	4 173 (100%)	7 246 (100%)

Numbers are patient numbers (% column).

Average drug use was lower in both groups than the WHO DDD, which consists of two daily inhalations for the medium and high doses of Seretide but two daily inhalations for the high dose and four daily inhalations for the medium dose of Symbicort. In the Seretide group, patients used an average of 1.38 to 1.95 inhalations for both doses. Symbicort users showed a very similar pattern; the patients used an average of 1.28 to 1.97 daily inhalations of the high-dose product and 1.74 to 2.49 inhalations of the medium-dose product. The latter showed the largest difference compared with the daily doses established by the WHO. In addition, it was observed that women used a significantly lower dose of Symbicort than men, both for high and medium doses (Table 3).

**Table 3:** Average daily inhalation of Symbicort Turbuhaler and Seretide Diskus in patients with asthma

	**High dose therapy**				**Medium dose therapy**			
	**Male**		**Female**		**Male**		**Female**	
**Age (years)**	**Seretide**	**Symbicort**	**Seretide**	**Symbicort**	**Seretide**	**Symbicort**	**Seretide**	**Symbicort**
18-30	1.38	1.28	1.36	1.19	1.30	1.90	1.95	1.74
31-40	1.34	1.97	1.28	1.47	1.36	1.97	1.32	1.78
41-50	1.61	1.62	1.34	1.48	1.33	2.07	1.36	2.03
51-60	1.46	1.66	1.37	1.56	1.39	2.40	1.33	2.22
61-70	1.51	1.87	1.47	1.67	1.44	2.38	1.42	2.27
>70	1.50	1.67	1.42	1.73	1.48	2.49	1.43	2.31

Table 4 summarises the results of the cost analysis. There were statistically significant differences for all medication-related cost items in the groups of patients using high-dose medications. The costs of the reliever medications (mean difference of 3.7 USD), the medications for exacerbation (mean difference of 15.9 USD) and the total medications (mean difference of 125.8 USD) were significantly lower in Symbicort users than in Seretide users. The cost of maintenance therapy (mean difference of 53.3 USD) for asthma was significantly lower for Seretide users. The costs of outpatient and inpatient care were similar between the groups.

**Table 4:** Average annual healthcare costs of asthma patients receiving medium- or high-dose Symbicort or Seretide Diskus therapy (USD)

**Group**	**Treatment**	**Number of patients**	**Expenditure (USD)**								
			**Maintenance therapy**	**Reliever medication**	**Exacerbation medication**	**Total medication**	Special* outpatient care	**Total outpatient care**	**Special† inpatient care**	**Total inpatient care**	**Total‡**
***High dose***	**Symbicort**	3 888	851.7	17.8	65.9	2140.2	42.7	220.1	59.2	760.2	3120.6
	**Seretide**	5 869	796.9	21.3	77.9	2540.3	43.1	228.6	75.2	786.6	3555.6
**raw difference (Sym vs Ser)**			54.8	−3.6	−12.0	−400.1	−0.4	−8,5	−16.0	−26.4	−435.0
**adjusted§ difference**			53.3	−3,7	−15.9	−125.8	−0.5	−9.2	−14.1	−11.4	−230.9
**95% CI of the adjusted difference**			25.1; 81.6	−5.3; -2.0	−23.3; -8.5	−242.3; -9.2	−3.0; 1.9	−29.6; 11.3	−33.6; 5.4	−140.9; 118.1	−628.3; 166.5
**p-value**			0.001	<10^-3^	<10^-3^	0.04	0.7	0.4	0.1	0.9	0.2
***Medium dose***	**Symbicort**	11 651	575.8	11.6	43.3	1563.0	40.2	208.2	43.1	510.0	2281.2
	**Seretide**	6 391	575.3	13.5	40.5	1590.5	38.9	211.2	30.0	494.4	2296.1
**raw difference (Sym vs Ser)**			0.6	−1.9	2.7	−27.5	1.3	−3.0	13.1	15.6	−14.9
**adjusted§ difference**			0.6	−2.2	3.4	9.0	1.7	3.8	14.2	34.9	93.1
**95% CI of the adjusted difference**			−20.0; 21.2	−4.0; -0.5	−0.8; 7.6	−68.7; 86.6	0.2; 3.14	−12.3; 19.9	3.9; 24.4	−18.6; 88.3	−87.9; 274.15
**p-value**			0.96	0.02	0.1	0.8	0.03	0.6	0.01	0.2	0.3

CI: confidence interval.

* ICD code of the diagnosis justifying care is J45 or J96.

† the ICD code on which the main diagnosis is based or that for the main diagnosis justifying the care is J45 or J96.

‡ total costs of drugs, both outpatient and inpatient care.

§ adjusted for age and gender.

Although the daily therapeutic costs calculated from the WHO DDD should be much lower for Seretide users, we did not find a difference in the actual costs of the maintenance therapy in patients using medium-dose medications. The cost of reliever medications in Symbicort users was significantly lower, by 2.2 USD, than in Seretide users. No differences were found in the costs of the medications for exacerbation or in total drug costs.

The outpatient and inpatient care costs for asthma were significantly lower in the adjusted analysis in the Seretide group. No significant differences were found between the two groups in terms of total medical costs.

## Discussion

In our study we evaluated whether real-world savings could be achieved by the National Health Insurance Fund with the current practice of therapeutic reference pricing in the R03AK06 ATC group and, if so, whether the therapeutic referencing methodology was appropriate. In our case we did not need to apply specific methodological techniques to separate the impact of TRP from GRP, as at the time of our analysis the R03AK06 ATC group did not include any generic products.

In the groups studied, patients used smaller doses of the drugs than recommended by the WHO DDD. Differences between the WHO DDD and prescribed daily doses were previously reported in several therapeutic areas [15,16]. Because the basis for therapeutic referencing is the daily therapy cost according to the WHO DDD, our analysis provides evidence for the limitations of this method in asthma. No relationship was found between the daily therapeutic costs calculated by means of the NHIF methodology and the real-world costs of actual drug use. Therefore, in our presented case the expected lower daily therapeutic costs did not exist in practice.

Our analysis also showed that therapeutic referencing based solely on the daily therapeutic costs in the R03AK06 group did not lead to a reduction of expenditure for the payer. TRP might even generate additional expenditures, as in addition to the costs calculated in this analysis, costs related to a switch to another treatment should also be taken into account. When patients change inhalation devices, they need to learn how to use the new device. Moreover, switching to a new inhalation device may reduce compliance due to the individual preferences of the patients, which may eventually lead to decreased effectiveness [17,18].

In interpreting the results, it should be taken into account that Seretide doses can only be increased by switching from the medium dose to the high dose; consequently, these cases were excluded from this study. The doses for Symbicort, on the other hand, can be adjusted in a different way because a daily dose of 2x1 inhalations can be increased to 2x2 inhalations. Therefore, an appropriate comparison for some of the patients using a medium dose of Symbicort would have been high-dose Seretide, which is also supported by the fact that daily inhalations of the medium dose of Symbicort were relatively high compared with the medium dose of Seretide. The higher daily inhalations of Symbicort may also be explained by the Symbicort SMART® indication. Although an analysis that took into account this dose increase could have been performed on individual patient data, we only had access to aggregated administrative financing data in this study.

Nevertheless, although this analysis followed a conservative approach with regard to Symbicort, NHIF still could not expect any real-world savings by referencing the WHO DDD.

This study also shows that administrative financial data support the clinical data, which suggest a decreased need for relievers and exacerbation treatments if patients are treated with Symbicort. This proves that differences in the indications cannot be disregarded during the process of referencing and that different indications should not be treated in the same way.

Our study has several limitations. Although the inclusion criteria guarantee that SYT and SED were prescribed for patients with asthma indication and not COPD, coding errors could not be excluded due to the retrospective nature of our analysis.

Medications for exacerbations could also be prescribed for non-asthma-related events. Similarly, outpatient and inpatient episodes could also be related to other comorbidities, so conclusions related to these outcomes have limited generalisability.

Confounding factors may bias our results. Theoretically, the different characteristics between the two study groups may partially explain the differences in costs. The effects of confounding could have been controlled by a multiple regression analysis. For such analyses, however, individual patient data would be required. According to the NHIF, however, such data could not be released for research purposes, not even in an anonymous form. Nevertheless, we used an adjustment method based on the aggregated data to control for age and gender.

## Conclusion

Cost-containment initiatives by payers may influence clinical decisions. TRP for inhalation asthma drugs raises special concern, because of differences in the therapeutic profile of pharmaceuticals and the lack of proven financial benefits after exclusion of the effect of generic price erosion. Our findings indicate that the presented TRP approach of asthma medications based on the daily therapeutic costs according to the WHO DDD does not result in reduced public healthcare spending in Hungary.

Further analysis is required to show whether TRP generates additional expenditures by inducing switching costs and reducing patient compliance. Potential confounding factors may limit the generalisability of our conclusions.

## Abbreviations

ATC, Anatomical Therapeutic Chemical Classification System; DDD, Defined Daily Dose; DOT, Days of Treatment; GDP, Gross Domestic Product; HUF, Hungarian Forint; NHIF, Hungarian National Health Insurance Fund; OECD, Organisation for Economic Co-operation and Development; PPP, Purchasing Power Parity; SYT, Symbicort Turbuhaler; TRP, Therapeutic Reference Pricing; USD, US Dollar; WHO, World Health Organization.

## Competing interests

The authors declare that they have no competing interests.

## Author’s contribution

ZK: study concept and design, interpretation of data, manuscript writing, guarantor.ZsAT: statistical input into study design, acquisition of data, statistical analysis, manuscript revision.ZB: medical revision of study design, interpretation of data, manuscript revision.ZV: epidemiological input into study design, data analysis, review of results, interpretation, manuscript writing. All authors read and approved the final manuscript.

## Pre-publication history

The pre-publication history for this paper can be accessed here:

http://www.biomedcentral.com/1471-2466/12/35/prepub
